# Preclinical therapeutics *ex ovo* quail eggs as a biomimetic automation-ready xenograft platform

**DOI:** 10.1038/s41598-021-02509-3

**Published:** 2021-12-02

**Authors:** Samuel V. Rasmussen, Noah E. Berlow, Lisa Hudson Price, Atiya Mansoor, Stefano Cairo, Sandra Rugonyi, Charles Keller

**Affiliations:** 1 Children’s Cancer Therapy Development Institute, 12655 SW Beaverdam Rd West, Beaverton, OR 97005 USA; 2 Xentech, Evry, France; 3 Department of Biomedical Engineering, Oregon Health and Science University, Portland, OR 97239 USA

**Keywords:** Cancer models, Assay systems, Chemotherapy

## Abstract

Preclinical cancer research ranges from in vitro studies that are inexpensive and not necessarily reflective of the tumor microenvironment to mouse studies that are better models but prohibitively expensive at scale. Chorioallantoic membrane (CAM) assays utilizing Japanese quail (*Coturnix japonica*) are a cost-effective screening method to precede and minimize the scope of murine studies for anti-cancer efficacy and drug toxicity. To increase the throughput of CAM assays we have built and optimized an 11-day platform for processing up to 200 quail eggs per screening to evaluate drug efficacy and drug toxicity caused by a therapeutic. We demonstrate ex ovo concordance with murine in vivo studies, even when the in vitro and in vivo studies diverge, suggesting a role for this quail shell-free CAM xenograft assay in the validation of new anti-cancer agents.

## Introduction

Pediatric cancer has historically limited new drug development as demonstrated by only 10 new agents earning primary childhood cancer FDA approval since 1978. To develop new therapies for rare disease, a cost-effective workflow from basic science target identification to preclinical research and then to clinical investigation is needed. Current preclinical research approaches move from in vitro studies to in vivo murine models; however, most of the time drug response data obtained from in vitro assays fail to be confirmed in vivo. As mouse studies are sometimes prohibitively expensive and time-consuming, taking tens of thousands of dollars and 10 or more weeks to complete, it is crucial to develop innovative cost and time-effective processes to improve the selection of anti-cancer agents to be prioritized for preclinical mouse studies Here we propose a re-examined and optimized the shell-free quail chorioallantoic membrane assay (CAM) as a precursor to mouse preclinical studies^1,2^.

CAM assays have traditionally utilized chicken (chick) or quail eggs, with chick being used more commonly. Chick CAM models have been employed for the study of angiogenesis, tumor growth and metastasis^3–5^. The CAM is a membrane formed by the fusion of the chorion and allantois membrane on embryonic day 5–6 (e5–6)^6^. The membrane will attach to the inside of the eggshell allowing respiration and calcium extraction for the growing embryo. The methods for culturing embryos fall into two categories: in ovo, whereby a small hole cut into the shell gives access to the embryo (for example, the studies performed by Sidney Farber in 1962^5^), or ex ovo with the embryo transferred to a cell culture plate and grown separate from the shell. The chick methods of in ovo incubation, while effective for chick survival, are time consuming and cannot be scaled because of the need for a skilled operator^7^. For ex ovo approach, another group has reported a culture method for Japanese quail that is novel but also time intensive and unsuitable for automation in multi-well plates^4,8^. Parenthetically, too, the Japanese quail (*Coturnix japonica*) genome was sequenced in 2016 and is publicly available.

Approaches to using the avian eggs and/or CAM assays have had preclinical drug administration (dosing) pharmacokinetic challenges, but in recent years the application of drugs into the quail or chick has been approached by topical and intravenous injection^9^. To circumvent systemic drug administration, drug can be admixed with tumor cells in an extracellular matrix that is applied to the quail CAM^1,3^.

CAM experiments have been conducted on a variety of cancers, but to our knowledge no medium- or high-throughput methods have been reported for either adult or pediatric cancers. Herein, we present an approach to increased throughput of CAM assays in the preclinical prioritization of anti-cancer compounds via an automation-ready 6-well plate format.

## Results

### Egg cracking and viability

**Figure 1:**
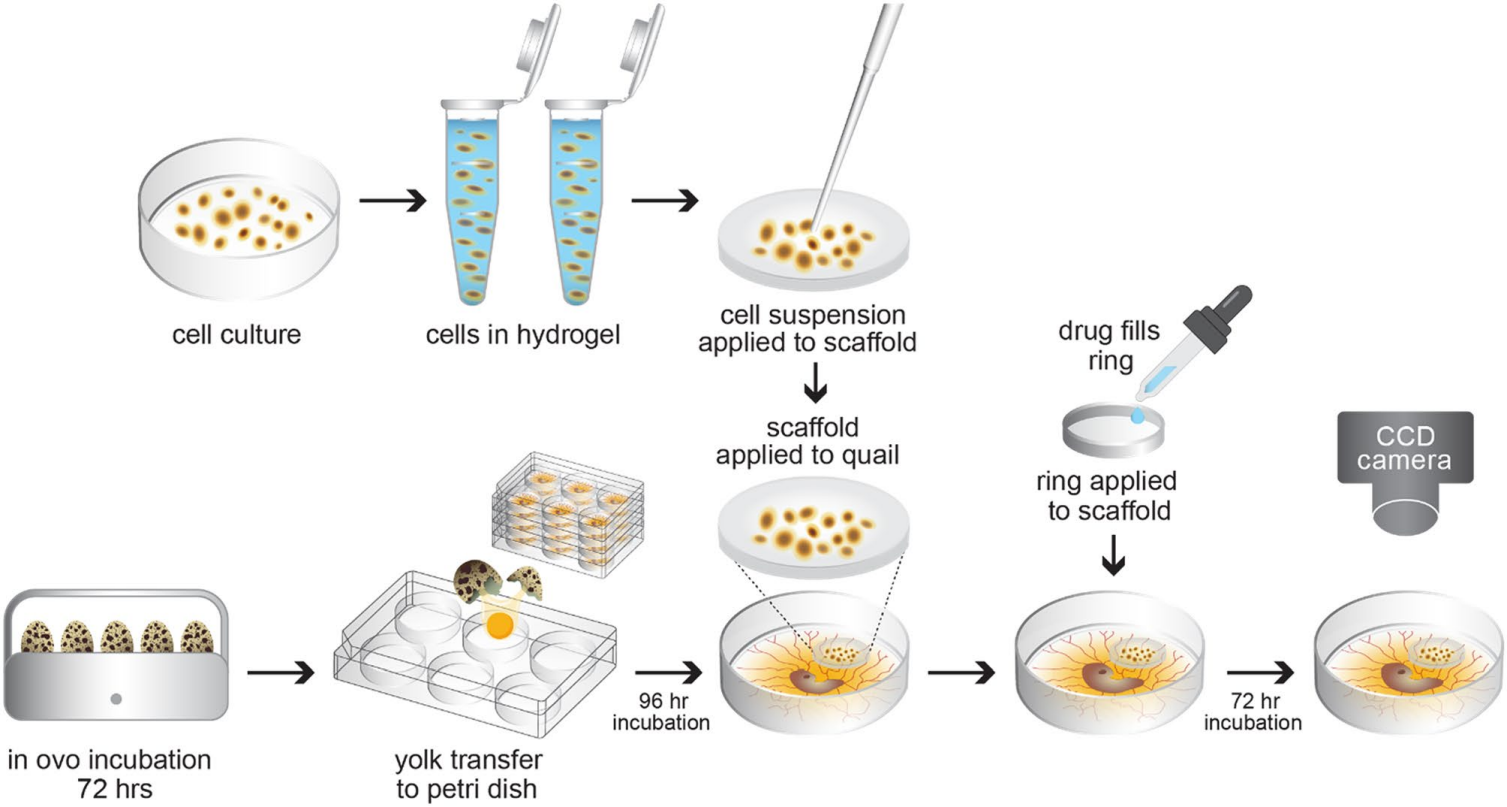
Schematic for the quail egg drug screening assay. The quail eggs are incubated for 72 h and then the embryos are transferred to six-well plates. The six-well plates are incubated for 96 h then any non-viable egg contents are removed. During the quail egg incubation, tumor cells are cultured. After the 96 h incubation the tumor cells are suspended in hydrogel, added to a 3D scaffold, and applied to the chorioallantoic membrane. A 9 mm ring is placed onto the 3D scaffold and then filled with P-10 osmotic beads soaked and equilibrated in the therapeutic compound. The quail is then incubated for 72 h. To analyze drug efficacy on tumor cells, luciferin is added to the scaffold and then imaged by a CCD camera to determine the total xenograft cell viability. Copyright holder Children’s Cancer Therapy Development Institute.

**Figure 2:**
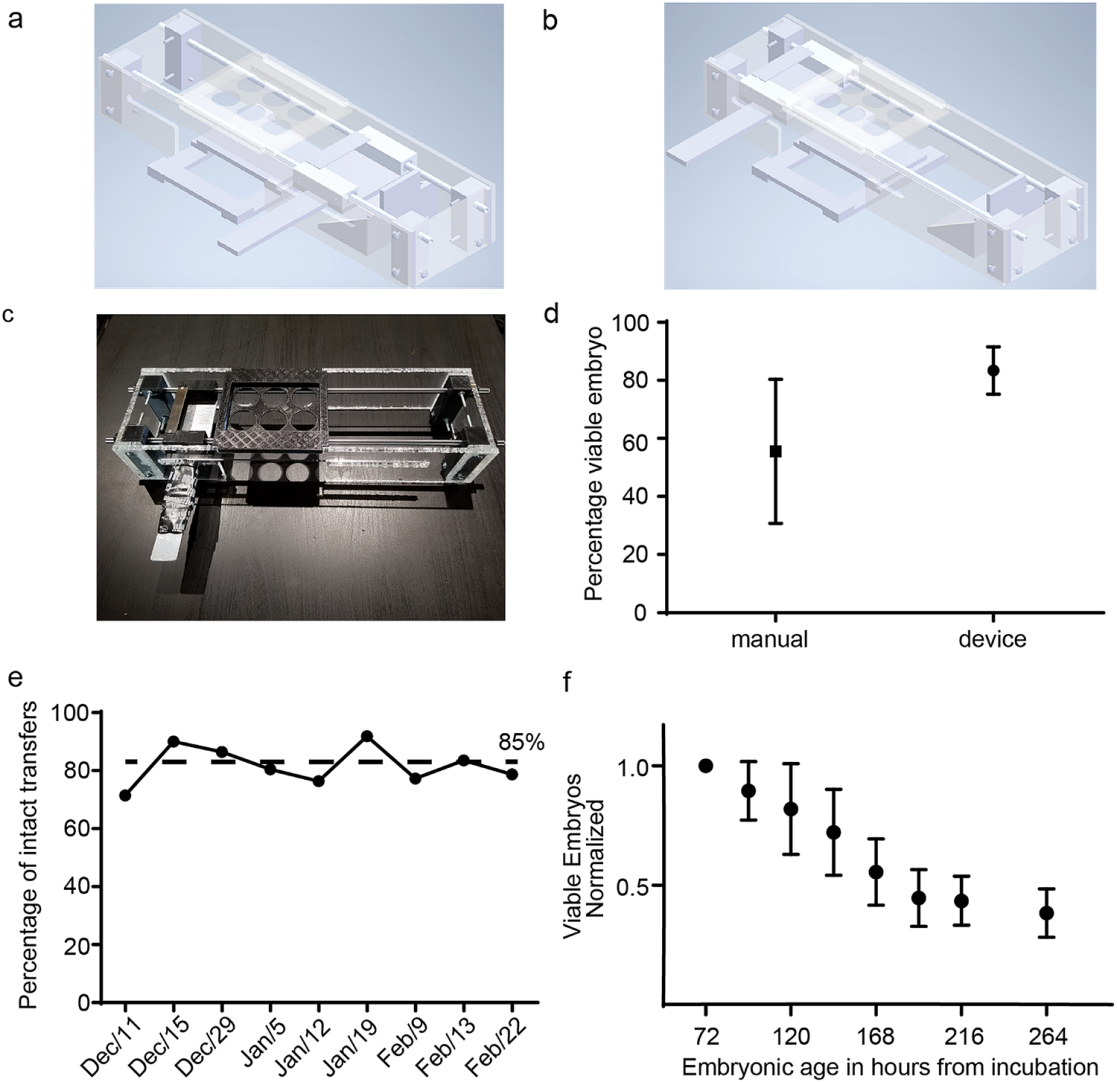
Embryo Transfer to multi-well plates. (**a**,**b**) CAD drawing of the quail egg opener in the before and after positions for opening quail eggs. (**c**) The blade collision device to open the quail eggs and transfer them to the six-well plate. (**d**) The quail batches used for the device method varied between 40 and 75 quail eggs per batch with 8 batches done. Three batches were used for the manual forceps method had between 32 and 75 quail eggs each with 3 batches done, error bars are standard deviation. (**e**) Number of viable egg yolks transferred to six-well plates weekly over a period of 9 weeks. (**f**) The age vs survival of the quail embryos used to choose the experiment start date error bars are standard deviation.

**Figure 3:**
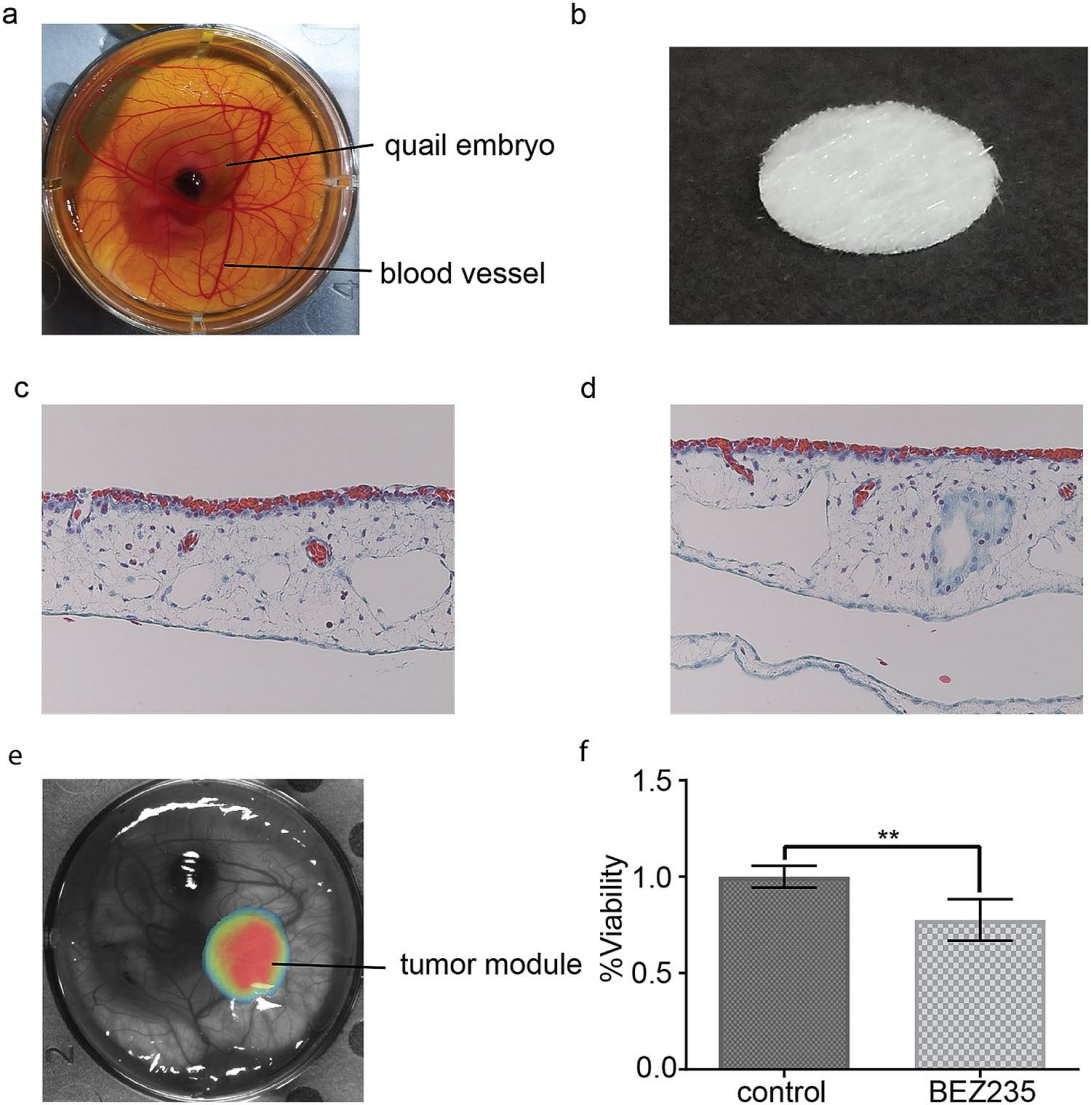
Quail xenograft assay. (**a**) a quail embryo at e7. (**b**) An unused 3D scaffold 9.5 mm in diameter for use as a tumor module. (**c**,**d**) Cross sections of the CAM. (**e**) representative pseudo-colorized image of viable, luciferase-expressing tumor cells 72 h after xenografting on the CAM. (**f**) The assay used murine rhabdomyosarcoma cells treated with BEZ235 (n = 6) or vehicle control (n = 4). **, significance p = 0.0048.

A schematic of the shell-free quail CAM assay is presented in Fig. 1. A rate-limiting step is the transfer of egg contents with the yolk intact to a multi-well plate. A simple apparatus to process 6 eggs at a time is shown in Fig. 2a–c. The total time required to conduct the transfer from in ovo to ex ovo for 100 eggs is approximately 2 h and 10 min. This approach has intact-yolk transfer rate of 85% on average as shown in Fig. 2d, with egg content transfer to multi-well plates is consistently achieved as shown in Fig. 2e. A second challenge was to determine the stage of egg development with the least spontaneous embryo death. From our studies, embryo survival decreases to 40–50% survival by embryonic day 10 with most of the losses between embryonic day 4 and day 7 (Fig. 2f); therefore, day 7 (e7) (Fig. 3a) was identified as the best timepoint for tumor xenografting.

### Validation of the reporter systems used

We next confirmed bioluminescence of tumor cells using a commercially-available luciferase-red fluorescent protein (RFP) lentivirus reporter system (Supplementary Fig. 1). The luminescence was linear with the tumor cell counts of xenograft-ready “modules” consisting of tumor cells embedded in hydrogel on a fiberglass scaffold (Supplementary Fig. 1). As a cautionary note, for the HepG2 cell line the luminescence and cell number were linear in our range employed but became non-linear at 4 × 10^6^ cells per tumor module (Supplementary Fig. 1). A transgenic rhabdomyosarcoma cell culture (U48484^2^) demonstrated a linear relationship between luminescence and tumor cell number for the range of 1 × 10^5^ and 1.25 × 10^6^ cells per module (data not shown). These ranges can be considered narrow however they provide an accurate linear range to compare and are bright enough to ensure visualization by bioluminescence.

### Ex ovo efficacy testing at single drug concentrations

We initially validated our system using the dual PI3K/mTOR inhibitor BEZ235 was tested on the rhabdomyosarcoma cell culture U48484^2^. A 3D scaffold (Fig. 3b) was used for the tumor module which was supported by the capillary-rich CAM (Fig. 3c,d). We tested BEZ235 at 500 nM in the hydrogel volume (Fig. 3e) and had an observable 20% reduction in U48484 tumor module bioluminescence (tumor cell viability) as shown in Fig. 3f. We are cautious to note, however, that the efficacy of BEZ235 could have been decreased by diffusion into the CAM, which led us to pursue a drug depot/osmotic drug release approach described below.

### In vitro and ex ovo efficacy testing across a drug concentration range

**Figure 4:**
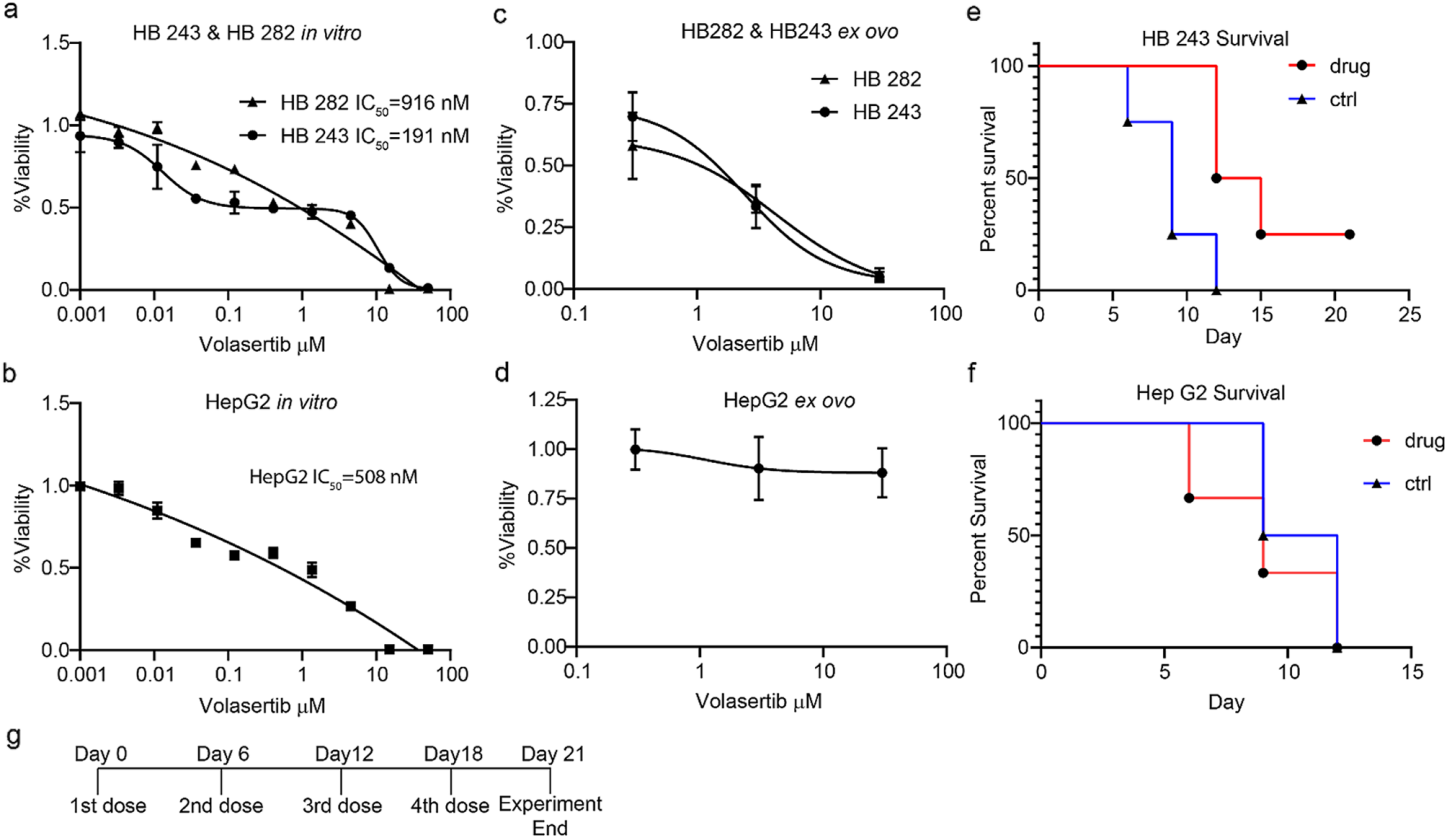
Comparison of in vitro, quail egg ex ovo xenograft and mouse in vivo xenograft results. (**a**,**b**) In vitro drug curves, each point has n = 4 replicates. (**c**,**d**) For the ex ovo drug curves, each concentration has n = 4–6 replicates. (**e**) The murine xenograft survival analyses for HB243 (n = 4) defined an event occurrence as tumor volume equal or greater than 0.75 cc, and a significant difference observed between treatment and control groups with a p = 0.031 using the Mantel-Cox test. (**f**) The survival of HepG2 bearing mice (n = 3) using the same criteria did not have a difference between treatment and control groups with p = 0.685. (**g**) Shown is the experiment timeline for drug dosing in mouse xenograft studies.

A PLK inhibitor (volasertib) was selected for testing against a range of hepatoblastoma cell lines given that PLK1 is a proposed therapeutic target^10^. The canonical hepatoblastoma cell line HepG2 and patient-derived xenograft (PDX) explanted cell lines HB243 and HB282 were selected as contemporary, robust patient-derived comparators^11^. Volasertib was tested both in vitro and ex ovo against the cell lines across a concentration range. Previous in vitro studies^10^ and our own results show that HB282 and HepG2 both were least sensitive to volasertib (IC_50_ values 916 nM Fig. 4a and 508 nM in Fig. 4b, respectively), whereas HB243 is considered the most sensitive (IC_50_ 191 nM in Fig. 4a). For CAM xenografts of these cell cultures, drug delivery is different than in vitro because of CAM vessel washout and embryo drug metabolism; however, in these experiments drug exposure to the engraftment modules was kept constant by osmotic bead drug delivery. Given the small blood volumes of the quail (Supplementary Fig. 2) and the desire to automate drug testing, the osmotic bead drug delivery system was chosen and validated for consistent small-molecular research (Supplemental Fig. 3) as the most cost-effective method to have a defined drug exposure/drug delivery over time. Ex ovo, HB243 and HB282 were congruent to in vitro studies with a 50% tumor growth inhibition (ex ovo IC_50_) of 1455 nM and 1040 nM, respectively (Fig. 4c). On the other hand, HepG2 was insensitive at any concentration as shown in Fig. 4d in stark contrast to the in vitro studies.

### Murine in vivo xenograft study comparisons

In cell line-based mouse xenograft studies, HB243 and HepG2 had different responses to volasertib in vivo. HB243 caused a statistically significant reduction in growth relative to the control group as shown in Fig. 4e with the treatment mice surviving to the endpoint an average of 4.5 days longer than control (vehicle). HepG2 had no increase in survival in Fig. 4f with no significant difference between the volasertib-treated cohort and the control cohort. Volasertib resistance in HepG2 could not be attributed to *PLK* expression or ABC transporter (drug efflux) expression (Supplementary Table 1) as we did not find a clear pattern with ABC genes in HepG2 having a consistently higher expression than HB243 and HB282 however some such as ANCG2 were expressed more in HepG2.

### Ex ovo patient-derived xenografts

Because generation of patient-derived xenograft using immune-compromised host mice can take 2–7 or more months^12^, we tested whether flat sections of patient tumor would engraft on the CAM immune-tolerant platform (Supplementary Fig. 3). Tumor engraftment (vascularization) from a mouse PDX-derived explant from an Ewing sarcoma occurred within 24 h and viability was maintained for 96 h (Supplementary Fig. 3). Similar results were obtained with an autopsy-derived Ewing sarcoma specimen cryo-preserved, thawed, and engrafted on the CAM (Supplementary Fig. 3).

### Quail toxicity assay

**Figure 5:**
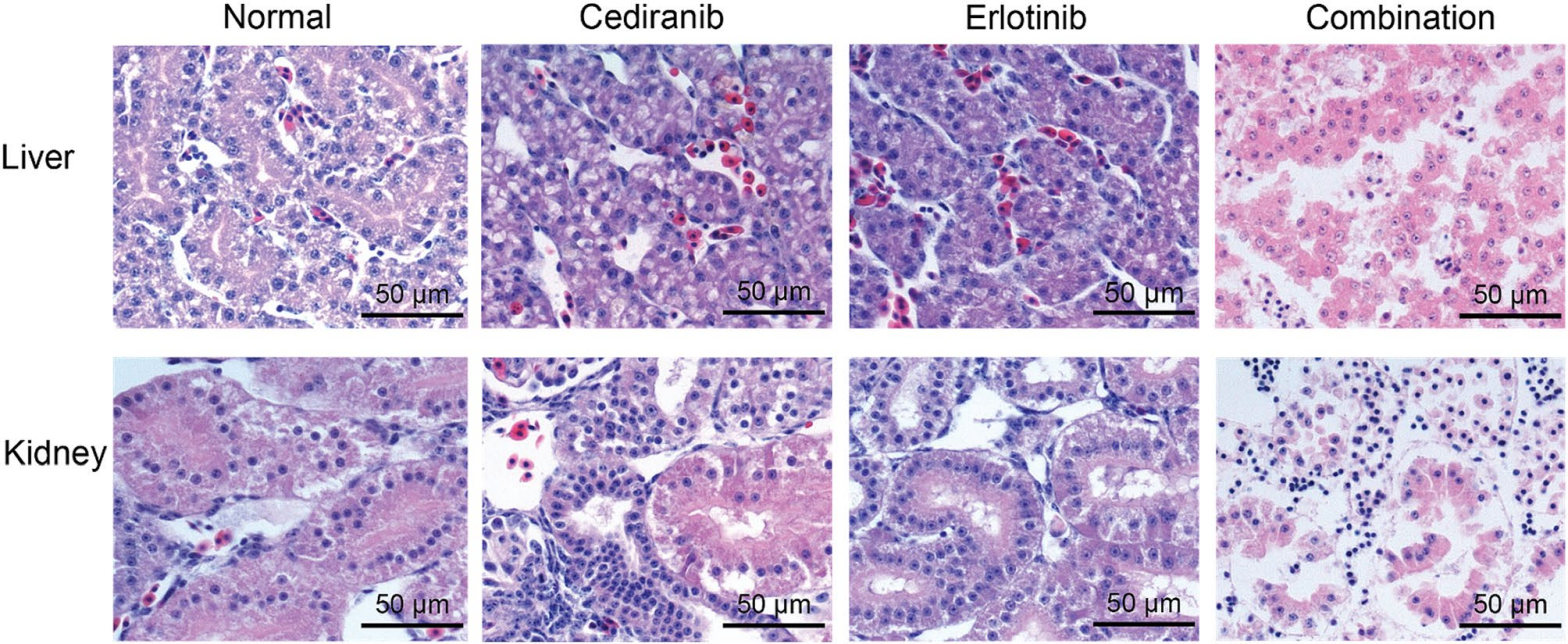
Histology slides stained with H&E of embryonic quail. Comparison of liver and kidney for e10 quail dosed with the human clinical C_max_ of cediranib (VEGFR and poly-kinase inhibitor) and/or erlotinib (EGFR inhibitor) revealing synergistic toxicity in liver and kidney.

The histology slides were examined by co-pathologist A.M and were found to have significantly more toxicity from the combination of cediranib and erlotinib than either drug alone. Synergistic toxicity was seen in the e10 quail embryo liver and kidney as shown in Fig. 5.

## Discussion

Herein we report methods for reliably and reproducibly performing ex ovo drug testing in shell-free quail CAM assay xenografts. We believe this platform brings us close to automation through mechanical systems for transferring egg contents to multi-well plates, and we demonstrate the feasibility of single concentration xenograft testing, or xenograft testing across a concentration range (*e.g.*, the ex ovo IC_50_). When culturing the quail ex ovo we noticed a majority of the die off between e4 and e7 most likely due to the shock of transferring the embryo to the six well plate. At e7 the chorioallantoic membrane had fully formed and providing a capillary rich surface to support the tumor module. We tested volasertib in mouse xenografts and compared the in vivo results to the ex ovo quail xenograft results and observed that HepG2 continued to be resistant in concordance with the ex ovo experiments and in contrast to the in vitro experiments. HB243 had a statistically significant reduction in growth in concordance to both the sensitivity of HB243 to volasertib in the ex ovo and in vitro experiments. The resistance of HepG2 to volasertib in vivo and ex ovo but not in vitro deserves further analysis. We conducted RNA sequencing that could not discern ABC transporters overexpression as the cause of resistance. Our validation studies were conducted solely for hepatoblastoma, but parallel remain to be done for other pediatric and adult cancers. The readiness in which patient xenografts engraft to the CAM is an exciting opportunity for further research.

Future directions will include addressing the pharmacokinetic considerations for efficacy and toxicity models in lieu of our described approach of hydrogel-based tumor modules as a single compartment model. Given the small blood volumes of the quail CAM, and the changing blood volumes from e7 to e11, serial micro-sampling approaches will require careful but worthwhile optimizations.

## Methods

### Quail preparation

All experiments were conducted in accordance with Children’s Cancer Therapy Development Institute policies and all relevant guidelines. *Coturnix japonica* eggs were purchased from Boyd’s Bird Company (Eagle Creek, OR) and PurelyPoultry (Fremont, WI), stored at 4 °C for 120 h and then incubated at 37 °C and 70% humidity for approximately 72 h. Quail eggs were opened by our mechanical device (Fig. 2a) and the embryos and white transferred to a six-well plate. Six-well plates were incubated for 96 h (embryonic day 7, e7) at which point unfertilized/non-viable egg contents were removed and viable quail used for assays.

### Cell lines

All cell lines were obtained as de-identified samples. HepG2 human liver carcinoma cells (ATCC, HB-8065) were transfected with lentiviral particles containing RFP, luciferase, and neomycin resistance following manufacturer’s instructions (cat#LVP677, Gentarget, San Diego, CA). Reporter-transfected cells were purified using 800 nM G418 antibiotic selection 24 h, flow sorted, then antibiotic selected again with the resulting cell line stably expressing RFP and luciferase. Cells were maintained in DMEM (cat#11990573,ThermoFisher Scientific, Waltham, MA) with 10% FBS (ThermoFisher Scientific, cat#10437036) and 1% penicillin–streptomycin (cat#15140122, ThermoFisher Scientific). HB282 was received from co-author Stefano Cairo [Xentech] and transfected with a lentiviral particle containing RFP, luciferase, and puromycin resistance following manufacturer’s instructions (cat#LVP674, Gentarget). HB282 followed the previously listed selection process but with a puromycin (cat#73342, Stemcell Technologies, Cambridge, MA) concentration of 2 µg/ml. HB282 was cultured in ADMEM, 10% FBS,1% penicillin–streptomycin, 1% L-glutamine. We received HB243 transfected with GFP and luciferase from co-author Stefano Cairo[Xentech]. Previously characterized U48484 murine alveolar rhabdomyosarcoma (aRMS) cells which stably express a luciferase reporter transgene were maintained in DMEM, 10% FBS, and 1% penicillin–streptomycin^2^.

### Luminescence calibration

All experiments were conducted in accordance with Children’s Cancer Therapy Development Institute policies and all relevant guidelines. To generate a standard curve for luminescence, 7 different cell densities ranging from 0 to 4 × 10^6^ of HepG2Glo were suspended in Hydrogel-c (cat#GS313, ESI-BIO, Alameda, CA) and 50 µl were added to 9.5 mm diameter sterilized fiberglass 3D mesh in Supplementary Fig. 1 (cat# SC-S510-0001, LenaBioscience, Atlanta, GA) according to manufacturer’s protocol in a 24 well plate. Two hundred µl of luciferin-d (cat#122,799, PerkinElmer, Waltham, MA) at 15 mg/ml diluted in PBS was added to each well, incubated for 10 min, and then imaged using a UVP Biospectrum 600 (Analytik JenaUS LLC, Upland, CA). Luminescence readings were processed using Prism 8.0 (Graphpad Software, San Diego, CA, https://www.graphpad.com/scientific-software/prism/). Luminescence calibration was performed in the same manner for U48484 mouse aRMS cells with the range of cells from 0 to 1.5 × 10^6^ cells per module.

### Patient-derived xenograft onto the CAM

A diagram of the procedure is presented in Supplementary Fig. 3 with all experiments carried out performed after receiving approval from the institutional animal care and use committee (IACUC) at Children's Cancer Therapy Development Institute. Samples were collected from patients who had given informed consent and enrolled in the CuReFAST tumor banking study approved of by the Children’s Cancer Therapy Development Institute’s Institutional Review Board (Advarra, protocol # cc-TDI-IRB-1). An Ewing sarcoma patient derived xenograft from a 23 year-old male surgically implanted to a NSG mouse at Jax laboratory (model ID TM01617) was removed from the mouse, encased in 2% agarose at 37 °C and sliced to 1 mm thick. The tumor slices were removed from the agarose and applied to an injury site on e7 quail embryos. Injury was created by placing a dry glass rod against the cam and carefully removing the glass rod. Twenty µl of Matrigel was added on top of the tumor slice to covering the slice to avoid any drying. The tumor engrafted for 96 h and was then removed and fixed in formalin. Samples were then sectioned and H & E stained at Oregon Health & Science University (OHSU) Histopathology Shared Resource.

### Chick to quail blood volume comparison

All experiments were conducted in accordance with Children’s Cancer Therapy Development Institute policies and all relevant guidelines. In order to develop pharmacokinetic approximations for the quail we compared literature sources for chicken embryo mass and blood volume growth to our measured quail embryo growth over the same Hamburger and Hamilton stages Supplementary Fig. 4. We assumed that the ratio of blood volume to body mass would be the same during the same growth stages.

### Quail tumor assay

All experiments were conducted in accordance with Children’s Cancer Therapy Development Institute policies and all relevant guidelines. U48484 mouse rhabdomyosarcoma cells were cultured, trypsinized and added to two different vials of hydrogel making a concentration of 10^6^ cells per 50 µl. BEZ235 (cat#S1009, Selleck Chemicals, Houston, TX) in a solution of 0.1% DMF (cat#TS-20673, Thermo Fisher Scientific) in PBS was added to 10^6^ U48484 mouse rhabdomyosarcoma cells mixed with 50 µl hydrogel for a final concentration of 500 nM BEZ235. For untreated eggs, 0.1% DMF in PBS was used as a control. 50 µl of cells/hydrogel/drug mixture were added to each scaffold and incubated for approximately 30–45 min at 37 °C and 100% humidity. As detailed above, a superficial injury was created on the chorioallantoic membrane and tumor module containing either drug or control was placed on top. Quail bearing tumor module models were incubated for 72 h. Add the end of the incubation, 100 µl of PBS containing 1.5 mg of luciferin-d (cat#122,799, PerkinElmer) was added to the 3D scaffold, incubated for 10 min in the dark, and bioluminescence was measured using a Fluorchem instrument (ProteinSimple, San Jose, CA). The quail were imaged with an 8 min exposure for total light emission.

### Quail dose response assay

All experiments were conducted in accordance with Children’s Cancer Therapy Development Institute policies and all relevant guidelines. The tumor modules for dose response assay were generated as described above but with 5 × 10^5^ cells per 50 µl. Drug was dissolved in DMSO for all levels to a final tumor module concentration of vehicle control, 0.3 µM, 3 µM, or 30 µM with n = 6. P-10 beads (cat#1,504,144, Bio-Rad, Hercules, CA) were soaked in PBS at the concentration of the modules for four hours at room temperature. Approximately 50 µl of bead solution was added to a 9.5 × 1.5 mm plastic ring placed on top of the tumor module forming a drug depot. The drug depot provided a constant source of drug keeping the tumor module at a constant concentration despite drug leaving the module for the quail, as shown in Supplementary Fig. 2. The IR820 had an exponential range between 0.001 and 1 µM as shown in Supplementary Fig. 2. The IR820 diffused through a tumor module at a constant rate and less than 10% of the IR820 diffused through as shown in Supplementary Fig. 2. The quail with tumor module models and drug depots were incubated for 72 h. Afterwards, 150 µl of 15 mg/ml luciferin (cat#122,799, Perkin Elmer) in PBS was added to the modules incubated for 10 min and then imaged on an IVIS Lumina (PerkinElmer) for between 15 s (for cell lines HepG2) or 1 min (for cell lines HB243 and HB282).

### In vivo mice experiment

All studies in mice were performed after receiving approval from the institutional animal care and use committee (IACUC) at Children's Cancer Therapy Development Institute and in accordance with ARRIVE Guidelines. Hep G2 and HB243 were suspended in Matrigel and injected into n = 10 eight week-old female nod scid gamma mice per cell line xenograft (Charles River, Hollister, CA, NOD.CB17*-Prkdc*^*scid*^/NCrCrl) with 2 × 10^6^ cells per 100 µl injection. The dosing schedule shown in Fig. 4g began after reaching 0.25 cubic centimeters in volume with mice that did not develop tumors excluded. Volasertib (cat# S2235, SelleckChem) was suspended in 3.75% DMSO and corn oil (cat# C8267, Sigma-Aldrich, St Louis, MO) at a concentration of 1.5 mg/ml with 100 µl injected intraperitoneally with mice chosen for the drug and control group randomly selected to minimize selection bias. The mice were imaged by intraperitoneal injection of luciferin-d according to manufacturer’s instructions. The tumors were measured using calipers every 3 days and the equation for volume was V = LxWxHx(π/6). At the study end or if the tumors reached 1.5 cc the tumors were removed and measured.

### Quail toxicity assay

All experiments were conducted in accordance with Children’s Cancer Therapy Development Institute policies and all relevant guidelines. Cediranib (Cat# S1017, Selleck Chemicals LLC, Houston, TX) and erlotinib (Cat# S7786) were purchased from Selleck Chemicals and reconstituted in dimethyl sulfoxide (DMSO) following the manufacturers recommendations and diluted to 10 mM stock concentration.

Fertilized Japanese quail eggs were incubated and plated as described previously. Quail were allowed to grow ex ovo in six-well plates until the quail had passed the patterning phase (e8 based on plating date). Each experimental arm was assigned n = 4 viable quail and treated with one of four experimental conditions: vehicle, cediranib, erlotinib, and cediranib + erlotinib. Dosages provided to quail were based on maximum clinically-achievable serum concentrations (C_max_) in human patients, specifically 42 ng/mL for cediranib^13^ and 1.3 µg/mL for erlotinib^14^. Stock concentrations were diluted in phosphate-buffered saline (PBS) to the respective target concentrations and to a final volume of 25 µL per agent. DMSO for vehicle was set at the DMSO volume used in the cediranib + erlotinib combination (8.4 µL DMSO).

Vehicle and diluted agents were subsequently applied dropwise to the quail chorioallantoic membrane. Quail were photographed at 0 h, 24 h, and 48 h. Remaining viable quail (n = 4 vehicle, n = 3 cediranib, n = 3 erlotinib, n = 4 combination) were sacrificed 48 h after dosing and fixed in 10% formalin for 24 h. Fixed quail were transported to the OHSU Histology core, paraffin embedded, sectioned in coronal orientation, and stained with hematoxylin and eosin. Stained images were analyzed by pathologist co-author A.M. for kidney and liver histopathology looking for signs of normal versus abnormal development (Fig. 5).

### Sequencing of samples

Each cell line was grown to 80% confluency, trypsinized, and snap frozen. RNA was extracted and sequenced by Beijing Genomics Institute (BGI, San Jose, CA). The quality of RNA prior to extraction was adequate for each cell line (DV < 200%). HiSeq 4000 was used for paired-end sequencing with 40 million reads for RNA. Raw FASTQ sequencing files were run through our in-house computational pipeline.

### Statistical analysis

For the murine in vivo xenograft study survival analysis, the tumor endpoint volumes for time-to-event (TTE) analysis were set at 0.75 cc and were collected to 1.5 cc. TTE was defined in days by selecting the day in which the tumor volume equaled or surpassed 0.75 cc. Animals that did not reach endpoint volume were assigned a TTE of 21 days for the HB243 analysis and a TTE of 12 days for the G2 analysis. The Kaplan–Meier survival plot represents the percentage of animals surviving at different time points during the study. These percentages were generated from the TTE data using GraphPad Prism 9.0 software (Graphpad Software, San Diego, CA, https://www.graphpad.com/scientific-software/prism/). Survival curve comparisons were analyzed using the Mantel-Cox and Gehan-Breslow-Wilcox tests (95% CI) through Graph Pad Prism 9.0 software. For the quail xenograft assay, significance was determined by an unpaired two-tailed t-test with Welch’s correction and a p-value less than 0.05 was considered statistically significant. Error bars represent ± standard error of the mean (SEM).

## Supplementary Information

Supplementary Information.
