# Supplementary material for: Preclinical therapeutics ex ovo quail eggs as a biomimetic automation-ready xenograft platform
Source: Sci Rep. 2021 Dec 2;11:23302. doi: 10.1038/s41598-021-02509-3 (PMC8639741; doi:10.1038/s41598-021-02509-3)
Supplement: Supplementary file 1 — Supplementary Information. [file 41598_2021_2509_MOESM1_ESM.pdf]

Preclinical Therapeutics *Ex Ovo*

Quail Eggs as a Biomimetic Automation-ready Xenograft Platform

Samuel V. Rasmussen, Noah E. Berlow, Lisa Hudson Price, Atiya Mansoor,
Stefano Cairo, Sandra Rugonyi, Charles Keller

Supplementary Information

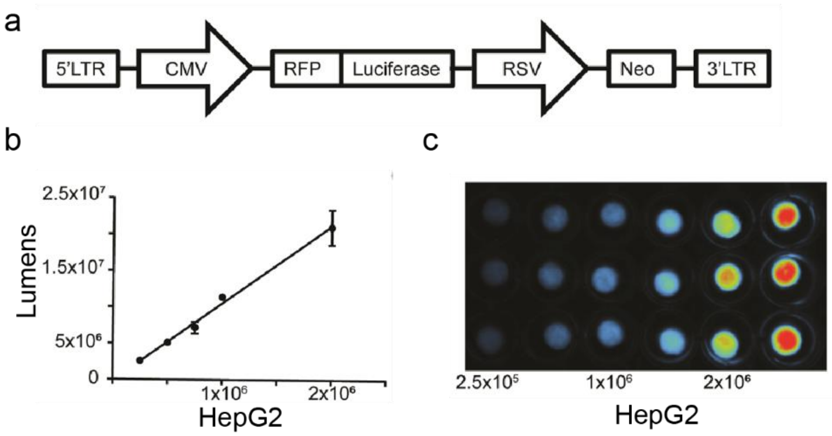

Supplementary Fig 1. Cell Number vs luminescence for HepG2Glo tumor modules. a) Lentiviral particle map used for transfecting HepG2. b) Linear relationship for bioluminescent vs HepG2 RFP cell number (R^2=0.927). c) The pseudo-colorized images of the multi-well plate holding the tumor modules with number of cells for the bioluminescence in panel b.

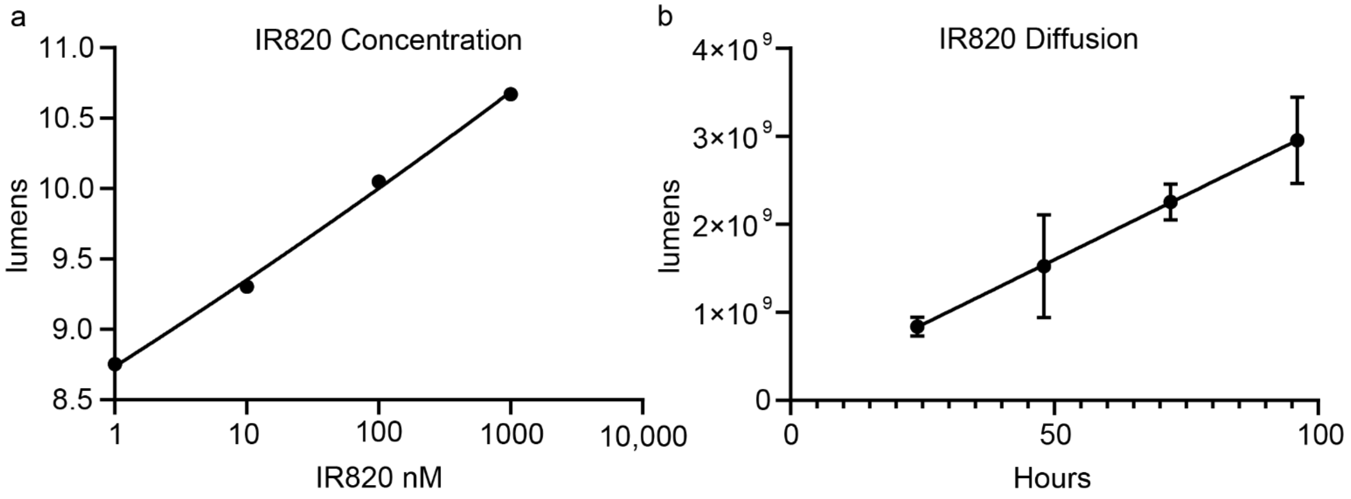

Supplementary Fig 2. The diffussion of IR820 small molecule dye from tumor modules. a) Tumor modules with varying concentrations of IR820 on a log-log scale and n=4 per concentration. b) The diffusion of IR820 into a multi-well plate from a tumor module with a P-10 drug depot beads applied atop of the scaffold.

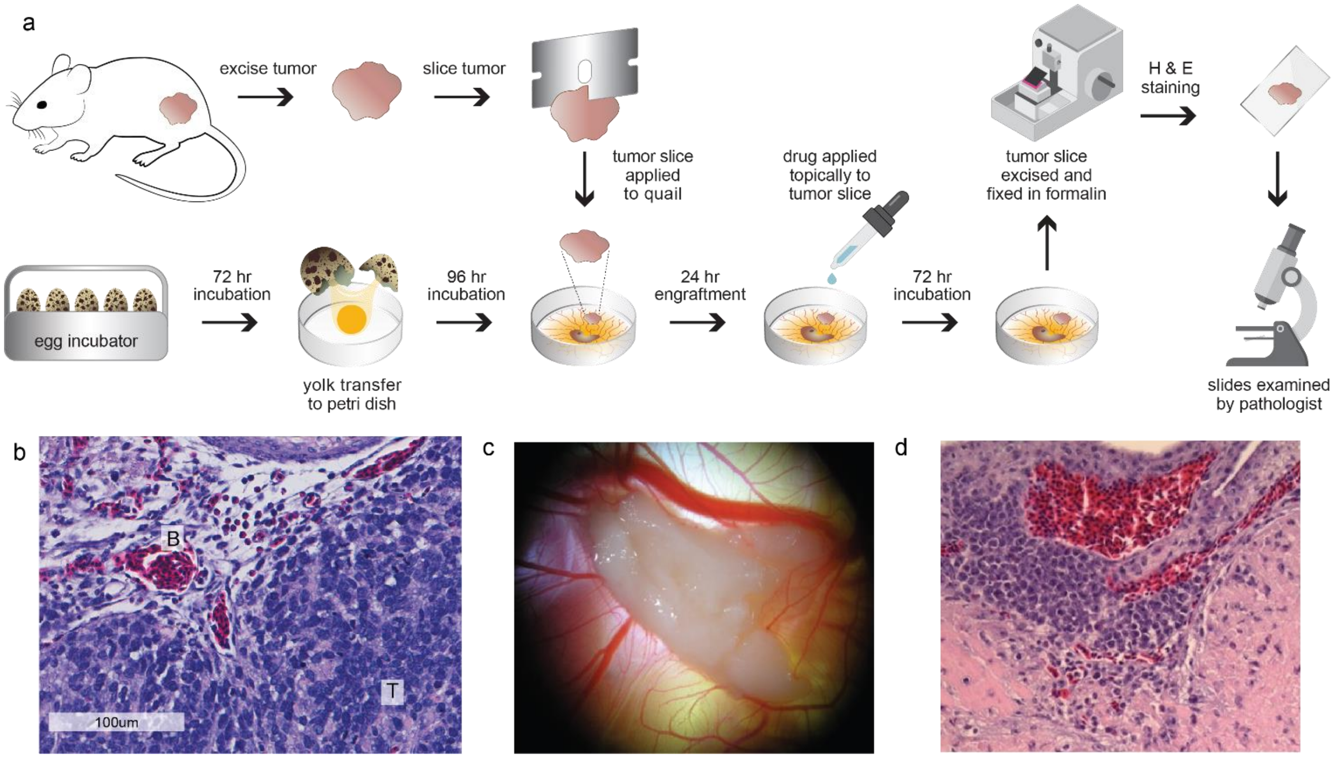

Supplementary Fig 3. *Ex ovo* patient-derived xenograft. Panel a is the process for xenografts copyright Children's Cancer Therapy Development Institute. Panel b is an Ewing sarcoma mouse PDX explant tumor engraftment. "T" is in the Ewing sarcoma tumor tissue. "B" represents nucleated red blood cells in the vasculature from the quail that has grown into the tumor. Panel c-d are autopsy-derived xenograft with vascularization.

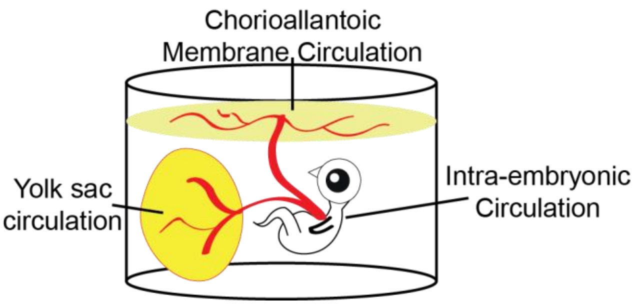

H&H Stage	Incubation Day Chick	Chick Embryo Mass (g)	Chick Blood Volume From Reference (mL)	Incubation Day Quail	Quail Embryo Mass Measured (g)	Quail Blood Volume Calculated (mL)
32	7	0.93	0.31	7	0.52	0.13
35	9	2.17	0.49	8	0.66	0.19
36	10	3.17	0.62	9	0.94	0.21
38	12	6.11	1.07	10	1.46	0.28
40	14	10.32	1.98	11	1.81	0.36
42	16	16.10	3.24	12	2.31	0.47

Supplementary Fig 4. Blood compartments of quail embryos and total blood volume for quail eggs. Quail embryo blood volumes calculated proportionally from the ratio of mass to blood volume of a chick for Hamburger and Hamilton developmental stages[1]. We assumed that the ratio of blood volume to body mass would be the same during the same growth stages.

Gene	Normal	HepG2	HB243	HB282
PLK1	1.45	69.26	47.88	31.45
PLK2	36.22	6.15	42.67	107.50
PLK3	7.99	5.39	2.14	6.67
ABCB1	10.52	37.14	49.07	157.00
ABCC2	59.14	86.07	123.00	84.96
ABCC3	26.53	13.28	39.74	29.49
ABCC6	27.99	3.07	5.28	2.41
ABCG2	14.64	9.79	3.34	5.89

Supplementary Table 1. mRNA expression levels in TPM for hepatoblastoma cell lines.

References

1. Mueller, C.A., Burggren, W.W. & Tazawa, H. *Sturkie's Avian Physiology*, (Elsevier, New York, 2015).
